# Methyl 4-*O*-benzyl-α-l-rhamno­pyrano­side

**DOI:** 10.1107/S1600536814007922

**Published:** 2014-04-16

**Authors:** Robert Pendrill, Lars Eriksson, Göran Widmalm

**Affiliations:** a Department of Organic Chemistry, Arrhenius Laboratory, Stockholm University, S-106 91 Stockholm, Sweden; b Department of Materials and Environmental Chemistry, Arrhenius Laboratory, Stockholm University, S-106 91 Stockholm, Sweden

## Abstract

In the title compound, C_14_H_20_O_5_, an inter­mediate in the synthesis of oligosaccharides, the glycosidic [H—C—O—C(H_3_)] torsion angle ϕ_H_ is 52.3° and the *exo*-cyclic [H—C—O—C(H_2_)] torsion angle θ_H_ is −11.7°. The hexa­pyran­ose ring has a chair conformation. In the crystal, mol­ecules are linked by O—H⋯O hydrogen bonds, forming chains propagating along [010]. Enclosed within the chains are *R*_3_^3^(12) ring motifs involving three mol­ecules. The chains are linked *via* C—H⋯π inter­actions, forming a three-dimensional network.

## Related literature   

For a description of l-rhamnose as part of polysaccharides, see: Ansaruzzaman *et al.* (1996 ▶); Marie *et al.* (1998 ▶); Säwén *et al.* (2012 ▶). For a description of syntheses in which the title compound has been used, see: Eklund *et al.* (2005 ▶); Handa *et al.* (1979 ▶). For the structure of rhamnosyl-containing tris­accharides, see: Eriksson & Widmalm (2012 ▶); Eriksson *et al.* (1999 ▶); Jonsson *et al.* (2006 ▶). For further related literature on l-rhamnose, see: Anderson & Ijeh (1994 ▶); Varki *et al.* (1999 ▶); Haines (1969 ▶); Herget *et al.* (2008 ▶); Olsson *et al.* (2005 ▶). For puckering analysis, see: Cremer & Pople (1975 ▶).
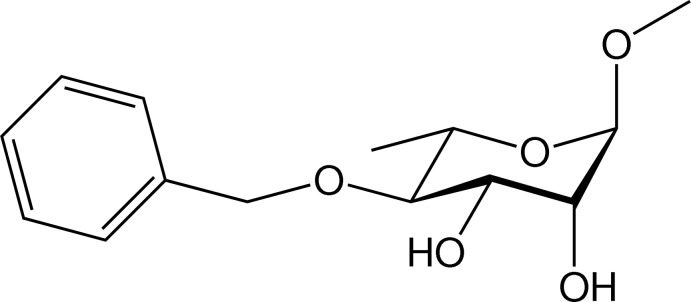


## Experimental   

### 

#### Crystal data  

C_14_H_20_O_5_
*M* *_r_* = 268.30
Orthorhombic, 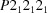

*a* = 6.5377 (1) Å
*b* = 9.1848 (2) Å
*c* = 23.2699 (5) Å
*V* = 1397.30 (5) Å^3^
*Z* = 4
Mo *K*α radiation
μ = 0.10 mm^−1^
*T* = 293 K
0.25 × 0.12 × 0.05 mm

#### Data collection  

Oxford Diffraction Xcalibur 3 with sapphire 3 CCD diffractometer
Absorption correction: multi-scan (*CrysAlis RED*; Oxford Diffraction, 2004 ▶) *T*_min_ = 0.921, *T*_max_ = 1.000
9540 measured reflections
1665 independent reflections
1407 reflections with *I* > 2σ(*I*)
*R*_int_ = 0.040

#### Refinement  

*R*[*F* ^2^ > 2σ(*F* ^2^)] = 0.035
*wR*(*F* ^2^) = 0.081
*S* = 1.00
1665 reflections
177 parameters
H-atom parameters constrained
Δρ_max_ = 0.14 e Å^−3^
Δρ_min_ = −0.13 e Å^−3^

### 

Data collection: *CrysAlis CCD* (Oxford Diffraction, 2004 ▶); cell refinement: *CrysAlis CCD*; data reduction: *CrysAlis RED* (Oxford Diffraction, 2004 ▶); program(s) used to solve structure: *SHELXS97* (Sheldrick, 2008 ▶); program(s) used to refine structure: *SHELXL97* (Sheldrick, 2008 ▶); molecular graphics: *DIAMOND* (Brandenburg, 2001 ▶); software used to prepare material for publication: *enCIFer* (Allen *et al.*, 2004 ▶).

## Supplementary Material

Crystal structure: contains datablock(s) I, rp1. DOI: 10.1107/S1600536814007922/su2708sup1.cif

Structure factors: contains datablock(s) I. DOI: 10.1107/S1600536814007922/su2708Isup2.hkl

CCDC reference: 996312

Additional supporting information:  crystallographic information; 3D view; checkCIF report

## Figures and Tables

**Table 1:** Hydrogen-bond geometry (Å, °) *Cg* is the centroid of the C41–C46 benzyl ring.

*D*—H⋯*A*	*D*—H	H⋯*A*	*D*⋯*A*	*D*—H⋯*A*
O2—H2*A*⋯O3^i^	0.82	2.00	2.813 (2)	172
O3—H3*A*⋯O5^ii^	0.82	2.05	2.799 (2)	151
C7—H7*C*⋯*Cg* ^iii^	0.96	2.89	3.652 (3)	137

Symmetry codes: (i) 
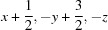
; (ii) 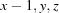; (iii) 
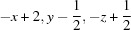
.

**Fig. 1.**
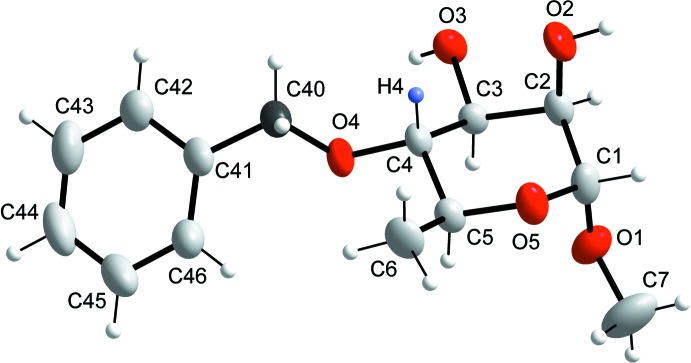
The molecular structure of the title molecule with atom labelling. Displacement ellipsoids are drawn at the 50% probability level. The long-range heteronuclear NMR coupling constant was measured beteen nuclei H4 (blue) and C40 (graphite).

**Fig. 2.**
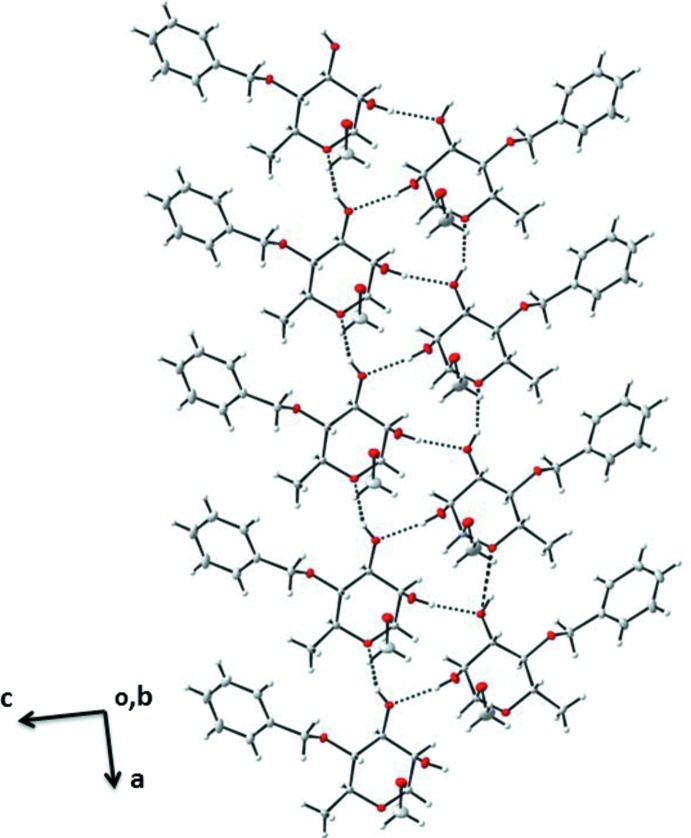
A partial view along the b axis of the crystal packing of the title compound. Hydrogen bonds are shown as dashed lines (see Table 1 for details).

## supplementary crystallographic information

### 1. Comment

Bacteria contain many different sugar residues (Herget *et al.*, 2008) in contrast to man where only a dozen monosaccharides are utilized in the formation of polysaccharides, glycoproteins and glycolipids [see Varki *et al.* (1999)]. In lipopolysaccharides *L*-rhamnose (6-deoxy-*L*-mannose) is often present as a sugar component, ranging from one residue per repeating unit, for example, as the terminal residue in the biosynthesized and polymerized oligosaccharide; consequently, it forms the side-chain residue in the O-antigen (Olsson *et al.*, 2005), which often has 10 – 25 repeating units. Alternatively, *L*-rhamnose can make up the O-antigen polysaccharide per se, as a homopolymer (Ansaruzzaman *et al.*, 1996).

The title compound, Fig. 1, has been used in the synthesis of a rhamnosyl-containing trisaccharide (Eklund *et al.*, 2005), the crystal structure of which was recently determined (Eriksson & Widmalm, 2012). The title monosaccharide is the methyl glycoside of α-*L*-rhamnopyranose and carries a benzyl protecting group at O4 in an ether linkage; the remaining two hydroxyl groups are unprotected and available for further synthetic modifications.

The glycosidic torsion angle defined by H1-C1-O1-C7, φ_H_ is 52.3° (Fig. 1). The *exo*-cyclic torsion angle defined by H4—C4—O4—C40, θ_H_ = -11.7°, shows an almost eclipsed conformation. The corresponding torsion angle in the crystal structure of 4-*O*-Benzyl-2,3-*O*-isopropylidene-α-*L*-rhamnopyranose was 36.8° (Eriksson *et al.*, 1999). Moreover, in the title compound the C4—O4—C40—C41 torsion is antiperiplanar and the benzyl ring plane deviates significantly from that defined by plane O4/C40/C41, with a dihedral angle of 54.85 (18)°.

The hexapyranose ring O5/C1-C5 has a chair conformation, with puckering parameters (Cremer & Pople, 1975) Q = 0.570 (2) Å, θ = 177.4 (2)° and φ = 11 (4)°. These puckering parameters reveal a ^1^C_4_ conformation close to the south pole, in contrast to another protected methyl α-*L*-rhamnopyranoside derivative carrying an isopropylidene group at O2 and O3 (Jonsson *et al.*, 2006).

In the crystal, molecules are linked via O—H···O hydrogen bonds, involving both hydroxyl groups, forming chains along the *a* axis (Table 1 and Fig. 2). They enclose 12-membered R^3^_3_(12) ring motifs. There are also C—H ··· π interactions present, between the C7 methyl group and the centroid of the (C41–C46) benzyl ring (Table 1), that link the chains forming a three-dimensional network.

The conformation of the *exo*-cyclic torsion angle (H4—C4—O4—C40) was analyzed by NMR measurements (see details in the archived CIF) of the long-range heteronuclear coupling constant between nuclei H4 and C40 using a J-HMBC experiment, which resulted in ^3^J_CH_ = 6.25 Hz. Interpretation of this coupling constant using the Karplus-type relationship ^3^J_C,H_ = 7.6 cos^2^θ - 1.7 cosθ + 1.6 (Anderson & Ijeh, 1994) leads to |θ_H_| = 26° when interpreted as a single conformation, *i.e.*, quite similar to the structure determined in the solid state. The corresponding torsion angle in the crystal structure of 4-*O*-Benzyl-2,3-*O*-isopropylidene-α-*L*-rhamnopyranose was 36.8° (Eriksson *et al.*, 1999).

### 2. Experimental

The synthesis of the title compound was performed according to a published procedure (Haines, 1969), where the rhamnosyl residue has the *L* absolute configuration. The title monosaccharide was crystallized at ambient temperature by slow evaporation from chloroform yielding colourless prismatic crystals. Spectroscopic data and details of the NMR measurements are given in the archived CIF.

### 3. Refinement

The OH and C-bound atoms were positioned geometrically and allowed to ride on their parent atoms: O-H = 0.82 Å, C-H = 0.98, 0.96 and 0.92 Å, for CH, CH_3_, and CH(aromatic) H atoms, respectively, with *U*_iso_(H) = 1.2*U*_eq_(C) and = 1.5*U*_eq_(O). In the final cycles of refinement, in the absence of significant anomalous scattering effects, Friedel pairs were merged and Δf " set to zero. The absolute configuration was set by the a priori knowledge of the absolute configuration of the starting reagent.

### Crystal data

**Table:**

C_14_H_20_O_5_	*F*(000) = 576
*M**_r_* = 268.30	*D*_x_ = 1.275 Mg m^−^^3^
Orthorhombic, *P*2_1_2_1_2_1_	Mo *K*α radiation, λ = 0.71073 Å
Hall symbol: P 2ac 2ab	Cell parameters from 4263 reflections
*a* = 6.5377 (1) Å	θ = 3.8–32.2°
*b* = 9.1848 (2) Å	µ = 0.10 mm^−^^1^
*c* = 23.2699 (5) Å	*T* = 293 K
*V* = 1397.30 (5) Å^3^	Prism, colourless
*Z* = 4	0.25 × 0.12 × 0.05 mm

### Data collection

**Table:**

Oxford Diffraction Xcalibur 3 with sapphire 3 CCD diffractometer	1665 independent reflections
Radiation source: Enhance (Mo) X-ray Source	1407 reflections with *I* > 2σ(*I*)
Graphite monochromator	*R*_int_ = 0.040
Detector resolution: 16.5467 pixels mm^-1^	θ_max_ = 26.4°, θ_min_ = 3.8°
ω scans at different φ	*h* = −3→8
Absorption correction: multi-scan (*CrysAlis RED*; Oxford Diffraction, 2004)	*k* = −11→11
*T*_min_ = 0.921, *T*_max_ = 1.000	*l* = −28→29
9540 measured reflections	

### Refinement

**Table:**

Refinement on *F*^2^	Secondary atom site location: difference Fourier map
Least-squares matrix: full	Hydrogen site location: inferred from neighbouring sites
*R*[*F*^2^ > 2σ(*F*^2^)] = 0.035	H-atom parameters constrained
*wR*(*F*^2^) = 0.081	*w* = 1/[σ^2^(*F*_o_^2^) + (0.0524*P*)^2^] where *P* = (*F*_o_^2^ + 2*F*_c_^2^)/3
*S* = 1.00	(Δ/σ)_max_ < 0.001
1665 reflections	Δρ_max_ = 0.14 e Å^−^^3^
177 parameters	Δρ_min_ = −0.13 e Å^−^^3^
0 restraints	Extinction correction: *SHELXL97* (Sheldrick, 2008), Fc^*^=kFc[1+0.001xFc^2^λ^3^/sin(2θ)]^-1/4^
Primary atom site location: structure-invariant direct methods	Extinction coefficient: 0.013 (2)

### Special details

**Table:**

Experimental. Spectroscoptic data for the title compound: ^1^H NMR (CDCl_3_, ppm, 298K, selected ^3^J_H,H_ values are given in parenthesis): H1 4.645(1.55); H2 3.903(3.49); H3 3.877(9.12); H4 3.333(9.52); H5 3.700(6.31); H6 1.352; H7 3.345; H40 4.738; H42,H46 7.355; H43,H45 7.360; H44 7.304; HO2 2.574(3.92); HO3 2.470(5.31). ^13^C NMR (CDCl_3_, ppm, 298K): C1 100.48; C2 71.20; C3 71.60; C4 81.77; C5 67.14; C6 18.14; C7 54.97; C40 75.11; C41 138.40; C42,C46 128.06; C43,C45 128.75; C44 128.12.NMR experiments were performed on a Bruker Avance III spectrometer operating at a ^1^H frequency of 700 MHz. The title compound was dissolved in chloroform-d and ^1^H and ^13^C resonances were referenced to internal TMS (δ = 0.0) and the solvent resonance (δ = 77.16), respectively. Resonance assignments were performed using standard experiments for oligosaccharides (Widmalm, G. (2007). NMR spectroscopy of carbohydrates and conformational analysis in solution. Comprehensive glycoscience, J. P. Kamerling, Ed., Elsevier, Oxford, Vol. 2, pp. 101–132) and measurement of the heteronuclear coupling constant was carried out by a J-HMBC experiment (Meissner, A. & Sørensen, O. W. (2001). *Magn. Reson. Chem.*39, 49–52) using two separate experiments with κ values of 59.0 and 99.0, respectively (Jonsson, K. H. M., Pendrill, R. & Widmalm, G. (2011). *Magn. Reson. Chem.*49, 117–124).
Geometry. All e.s.d.'s (except the e.s.d. in the dihedral angle between two l.s. planes) are estimated using the full covariance matrix. The cell e.s.d.'s are taken into account individually in the estimation of e.s.d.'s in distances, angles and torsion angles; correlations between e.s.d.'s in cell parameters are only used when they are defined by crystal symmetry. An approximate (isotropic) treatment of cell e.s.d.'s is used for estimating e.s.d.'s involving l.s. planes.
Refinement. Refinement of *F*^2^ against ALL reflections. The weighted *R*-factor *wR* and goodness of fit *S* are based on *F*^2^, conventional *R*-factors *R* are based on *F*, with *F* set to zero for negative *F*^2^. The threshold expression of *F*^2^ > σ(*F*^2^) is used only for calculating *R*-factors(gt) *etc*. and is not relevant to the choice of reflections for refinement. *R*-factors based on *F*^2^ are statistically about twice as large as those based on *F*, and *R*- factors based on ALL data will be even larger.

### Fractional atomic coordinates and isotropic or equivalent isotropic displacement parameters (Å^2^)

**Table:**

	*x*	*y*	*z*	*U*_iso_*/*U*_eq_	
C1	1.0867 (3)	0.5237 (2)	0.04789 (8)	0.0370 (5)	
H1	1.1807	0.5419	0.0159	0.044*	
C2	0.8931 (3)	0.6130 (2)	0.03845 (8)	0.0317 (5)	
H2	0.8232	0.5790	0.0037	0.038*	
C3	0.7515 (3)	0.5959 (2)	0.09008 (8)	0.0273 (4)	
H3	0.7109	0.4935	0.0933	0.033*	
C4	0.8644 (3)	0.6397 (2)	0.14447 (8)	0.0282 (4)	
H4	0.8990	0.7435	0.1432	0.034*	
C5	1.0597 (3)	0.5481 (2)	0.15042 (8)	0.0314 (4)	
H5	1.0212	0.4456	0.1549	0.038*	
O5	1.1851 (2)	0.56299 (17)	0.09964 (6)	0.0382 (4)	
C6	1.1946 (3)	0.5915 (3)	0.20013 (9)	0.0444 (6)	
H6A	1.3178	0.5347	0.1994	0.067*	
H6B	1.1234	0.5747	0.2356	0.067*	
H6C	1.2284	0.6929	0.1970	0.067*	
O1	1.0298 (2)	0.37637 (17)	0.04700 (6)	0.0458 (4)	
C7	1.1990 (5)	0.2788 (3)	0.04520 (14)	0.0819 (10)	
H7A	1.2799	0.2985	0.0117	0.123*	
H7B	1.1498	0.1804	0.0437	0.123*	
H7C	1.2813	0.2918	0.0790	0.123*	
O2	0.9419 (2)	0.76226 (15)	0.03268 (6)	0.0444 (4)	
H2A	0.9920	0.7768	0.0009	0.067*	
O3	0.5733 (2)	0.68189 (16)	0.08031 (6)	0.0355 (3)	
H3A	0.4761	0.6461	0.0975	0.053*	
O4	0.7372 (2)	0.60980 (15)	0.19307 (5)	0.0337 (3)	
C40	0.7222 (3)	0.7259 (2)	0.23403 (8)	0.0380 (5)	
H40A	0.6411	0.8049	0.2183	0.046*	
H40B	0.8573	0.7630	0.2430	0.046*	
C41	0.6222 (3)	0.6670 (2)	0.28745 (8)	0.0355 (5)	
C42	0.4478 (3)	0.7314 (3)	0.31025 (9)	0.0424 (5)	
H42	0.3947	0.8154	0.2937	0.051*	
C43	0.3531 (4)	0.6700 (3)	0.35777 (10)	0.0563 (7)	
H43	0.2358	0.7130	0.3727	0.068*	
C44	0.4302 (5)	0.5471 (3)	0.38299 (10)	0.0601 (7)	
H44	0.3630	0.5050	0.4141	0.072*	
C45	0.6085 (5)	0.4856 (3)	0.36203 (10)	0.0594 (7)	
H45	0.6650	0.4044	0.3799	0.071*	
C46	0.7013 (4)	0.5455 (3)	0.31468 (9)	0.0471 (6)	
H46	0.8205	0.5033	0.3006	0.057*	

### Atomic displacement parameters (Å^2^)

**Table:**

	*U*^11^	*U*^22^	*U*^33^	*U*^12^	*U*^13^	*U*^23^
C1	0.0251 (10)	0.0573 (15)	0.0288 (10)	−0.0006 (10)	0.0027 (9)	−0.0024 (9)
C2	0.0274 (9)	0.0430 (12)	0.0247 (9)	−0.0035 (10)	−0.0007 (8)	0.0023 (8)
C3	0.0220 (9)	0.0325 (10)	0.0275 (10)	−0.0004 (8)	−0.0010 (8)	0.0023 (8)
C4	0.0274 (9)	0.0345 (11)	0.0226 (9)	−0.0045 (8)	0.0032 (8)	0.0023 (8)
C5	0.0263 (9)	0.0400 (11)	0.0277 (9)	−0.0027 (9)	−0.0010 (8)	0.0033 (9)
O5	0.0216 (6)	0.0611 (9)	0.0320 (7)	−0.0031 (7)	−0.0010 (6)	0.0002 (7)
C6	0.0342 (11)	0.0628 (15)	0.0362 (11)	−0.0018 (11)	−0.0118 (10)	0.0040 (10)
O1	0.0394 (8)	0.0460 (9)	0.0520 (9)	0.0094 (8)	−0.0054 (8)	−0.0125 (7)
C7	0.0693 (19)	0.0734 (19)	0.103 (2)	0.0371 (18)	−0.0094 (18)	−0.0190 (18)
O2	0.0489 (9)	0.0499 (10)	0.0344 (8)	−0.0046 (8)	0.0109 (7)	0.0105 (7)
O3	0.0228 (7)	0.0481 (8)	0.0357 (8)	0.0034 (7)	0.0002 (6)	0.0070 (7)
O4	0.0351 (7)	0.0410 (8)	0.0250 (7)	−0.0071 (7)	0.0058 (6)	−0.0019 (6)
C40	0.0469 (12)	0.0375 (11)	0.0295 (10)	−0.0021 (11)	0.0033 (10)	−0.0013 (9)
C41	0.0449 (11)	0.0374 (12)	0.0244 (10)	−0.0076 (10)	−0.0002 (9)	−0.0050 (8)
C42	0.0451 (12)	0.0494 (13)	0.0326 (11)	−0.0021 (12)	−0.0008 (10)	−0.0062 (10)
C43	0.0519 (14)	0.0757 (18)	0.0414 (13)	−0.0124 (14)	0.0130 (12)	−0.0143 (13)
C44	0.0819 (19)	0.0642 (16)	0.0343 (12)	−0.0272 (17)	0.0159 (13)	−0.0018 (12)
C45	0.097 (2)	0.0465 (14)	0.0349 (12)	−0.0048 (15)	0.0069 (15)	0.0030 (10)
C46	0.0603 (15)	0.0441 (12)	0.0369 (11)	0.0019 (12)	0.0111 (11)	−0.0007 (10)

### Geometric parameters (Å, º)

**Table:**

C1—O1	1.404 (3)	C7—H7A	0.9600
C1—O5	1.412 (2)	C7—H7B	0.9600
C1—C2	1.524 (3)	C7—H7C	0.9600
C1—H1	0.9800	O2—H2A	0.8200
C2—O2	1.414 (2)	O3—H3A	0.8200
C2—C3	1.525 (3)	O4—C40	1.433 (2)
C2—H2	0.9800	C40—C41	1.505 (3)
C3—O3	1.426 (2)	C40—H40A	0.9700
C3—C4	1.519 (2)	C40—H40B	0.9700
C3—H3	0.9800	C41—C46	1.383 (3)
C4—O4	1.431 (2)	C41—C42	1.390 (3)
C4—C5	1.535 (3)	C42—C43	1.387 (3)
C4—H4	0.9800	C42—H42	0.9300
C5—O5	1.445 (2)	C43—C44	1.369 (4)
C5—C6	1.508 (3)	C43—H43	0.9300
C5—H5	0.9800	C44—C45	1.384 (4)
C6—H6A	0.9600	C44—H44	0.9300
C6—H6B	0.9600	C45—C46	1.373 (3)
C6—H6C	0.9600	C45—H45	0.9300
O1—C7	1.424 (3)	C46—H46	0.9300
			
O1—C1—O5	112.30 (17)	H6B—C6—H6C	109.5
O1—C1—C2	107.24 (16)	C1—O1—C7	113.7 (2)
O5—C1—C2	111.33 (16)	O1—C7—H7A	109.5
O1—C1—H1	108.6	O1—C7—H7B	109.5
O5—C1—H1	108.6	H7A—C7—H7B	109.5
C2—C1—H1	108.6	O1—C7—H7C	109.5
O2—C2—C1	110.35 (16)	H7A—C7—H7C	109.5
O2—C2—C3	108.14 (15)	H7B—C7—H7C	109.5
C1—C2—C3	109.60 (15)	C2—O2—H2A	109.5
O2—C2—H2	109.6	C3—O3—H3A	109.5
C1—C2—H2	109.6	C4—O4—C40	115.00 (14)
C3—C2—H2	109.6	O4—C40—C41	108.17 (15)
O3—C3—C4	112.53 (15)	O4—C40—H40A	110.1
O3—C3—C2	108.27 (14)	C41—C40—H40A	110.1
C4—C3—C2	109.53 (15)	O4—C40—H40B	110.1
O3—C3—H3	108.8	C41—C40—H40B	110.1
C4—C3—H3	108.8	H40A—C40—H40B	108.4
C2—C3—H3	108.8	C46—C41—C42	118.4 (2)
O4—C4—C3	108.97 (13)	C46—C41—C40	120.40 (19)
O4—C4—C5	107.88 (14)	C42—C41—C40	121.2 (2)
C3—C4—C5	109.51 (15)	C41—C42—C43	119.8 (2)
O4—C4—H4	110.1	C41—C42—H42	120.1
C3—C4—H4	110.1	C43—C42—H42	120.1
C5—C4—H4	110.1	C44—C43—C42	120.9 (2)
O5—C5—C6	105.68 (15)	C44—C43—H43	119.6
O5—C5—C4	110.25 (15)	C42—C43—H43	119.6
C6—C5—C4	114.22 (17)	C43—C44—C45	119.7 (2)
O5—C5—H5	108.8	C43—C44—H44	120.2
C6—C5—H5	108.8	C45—C44—H44	120.2
C4—C5—H5	108.8	C46—C45—C44	119.4 (3)
C1—O5—C5	114.51 (14)	C46—C45—H45	120.3
C5—C6—H6A	109.5	C44—C45—H45	120.3
C5—C6—H6B	109.5	C45—C46—C41	121.7 (2)
H6A—C6—H6B	109.5	C45—C46—H46	119.1
C5—C6—H6C	109.5	C41—C46—H46	119.1
H6A—C6—H6C	109.5		
			
O1—C1—C2—O2	−173.76 (14)	C4—C5—O5—C1	−57.5 (2)
O5—C1—C2—O2	63.0 (2)	O5—C1—O1—C7	−67.8 (2)
O1—C1—C2—C3	67.3 (2)	C2—C1—O1—C7	169.56 (19)
O5—C1—C2—C3	−55.9 (2)	C3—C4—O4—C40	−132.63 (17)
O2—C2—C3—O3	58.98 (19)	C5—C4—O4—C40	108.58 (18)
C1—C2—C3—O3	179.27 (15)	C4—O4—C40—C41	−168.12 (15)
O2—C2—C3—C4	−64.05 (19)	O4—C40—C41—C46	54.2 (2)
C1—C2—C3—C4	56.2 (2)	O4—C40—C41—C42	−124.9 (2)
O3—C3—C4—O4	65.15 (19)	C46—C41—C42—C43	−2.5 (3)
C2—C3—C4—O4	−174.41 (15)	C40—C41—C42—C43	176.6 (2)
O3—C3—C4—C5	−177.09 (14)	C41—C42—C43—C44	0.5 (3)
C2—C3—C4—C5	−56.64 (19)	C42—C43—C44—C45	2.2 (4)
O4—C4—C5—O5	174.32 (14)	C43—C44—C45—C46	−2.8 (4)
C3—C4—C5—O5	55.90 (18)	C44—C45—C46—C41	0.7 (4)
O4—C4—C5—C6	−66.9 (2)	C42—C41—C46—C45	1.9 (3)
C3—C4—C5—C6	174.69 (16)	C40—C41—C46—C45	−177.2 (2)
O1—C1—O5—C5	−62.6 (2)	C40—O4—C4—H4	−11.7
C2—C1—O5—C5	57.6 (2)	C7—O1—C1—H1	52.3
C6—C5—O5—C1	178.65 (18)		

### Hydrogen-bond geometry (Å, º)

Cg is the centroid of the C41–C46 benzyl ring.

**Table:**

*D*—H···*A*	*D*—H	H···*A*	*D*···*A*	*D*—H···*A*
O2—H2*A*···O3^i^	0.82	2.00	2.813 (2)	172
O3—H3*A*···O5^ii^	0.82	2.05	2.799 (2)	151
C7—H7*C*···*Cg*^iii^	0.96	2.89	3.652 (3)	137

Symmetry codes: (i) *x*+1/2, −*y*+3/2, −*z*; (ii) *x*−1, *y*, *z*; (iii) −*x*+2, *y*−1/2, −*z*+1/2.
